# The protective effect of 17 beta-estradiol on oxygen-induced retinopathy and its relation with the changes of malondialdehyde^☆^

**DOI:** 10.1016/S1674-8301(10)60022-X

**Published:** 2010-03

**Authors:** Hongbing Zhang, Naixue Sun, Houcheng Liang, Xianghua Xiao, Xianning Liu, Yani Wang

**Affiliations:** a Department of Ophthalmology, the Second Affiliated Hospital of Xi'an Jiaotong University, Xi'an, Shaanxi Province 710004, China.; b Eye Institute of Shaanxi Province and Xi'an Eye Hospital, Xi'an, Shaanxi Province 710002, China.

**Keywords:** oxidative stress, estradiol, receptor, oxygen induced retinopathy

## Abstract

**Objective:**

Retinopathy of prematurity is becoming obvious with the improvement of neonatal ambulance. However there is still not a good treatment. The present study is to observe the effect of 17 beta-estradiol (E2) on oxygen-induced retinopathy (OIR), and explore the relationship between the changes of avascular area and malondialdehyde (MDA) in retina.

**Methods:**

Newborn oxygen-exposed mice underwent subcutaneous injections of different dose of E2 (0.1 µg, 1.0 µg, 10.0 µg ), tamoxifen or phosphate buffered saline (PBS; controls)everyday from post-natal day (p)7 to p17. At p17, retinal flat mounts were scored for the percentage of avascular/total retinal area, and pathological changes during revascularization. The MDA concentration in the retina was determined also. In the most efficacious E2 group (10.0 µg), 100.0 µg tamoxifen was also administered, and the percentage of capillary-free/total retinal area determined, and the retinal malondialdehyde concentration assayed.

**Results:**

The mean percentage of capillary-free area over total retinal area was 0(PBS, in room air), 34.197±1.301(PBS, in hyperoxia), 23.685±0.407 (0.1 µg E2), 14.648±0.355 (1.0 µg E2), 4.693±0.450 (10.0 µg E2) and 32.240±0.654 (10.0 µg E2 +100.0 µg tamoxifen). The difference was significant (*F* = 2778.759, *P* < 0.01), and the difference between any two groups were also significant (all *P* value were less than 0.01). The predilection of tufts and clusters during revascularization was mainly aggregated in zones 2 and 3, but the difference of retinal neovascular clusters and tufts in fourth zone among different groups were significant [clusters (*F* = 44.719, *P* < 0.01) *vs* tufts (*F* = 39.997, *P* < 0.01)]. The mean MDA concentration were 0.711±0.037(PBS, in room air), 2.084±0.066 (PBS, in hyperoxia), 1.829±0.091(0.1 µg E2), 1.152±0.067(1.0 µg E2), 0.796±0.027(10.0 µg E2), 1.988±0.049(10.0 µg E2 +100.0 µg tamoxifen) (*F* = 628.103, *P* < 0.01). The difference between any two groups were also significant (all *P* value were less than 0.05). The close relation between the percentage of avascular/total retinal area and MDA concentration was also verified (*r* = 0.981, *P* < 0.01).

**Conclusion:**

Oxidative stress responses play a pivotal role in OIR, by means of receptor pathway. E2 can alleviate oxidative stress reaction, and thus ameliorate the severity of oxygen induced retinopathy.

## INTRODUCTION

Retinopathy of prematurity (ROP), a kind of vasoproliferative retinal disease associated with premature and low body weight infants, is becoming more and more serious with the improvement of neonatal ambulance[1]. Current treatments consist of close monitoring of oxygen saturation levels, peripheral retinal ablation by cryotherapy or laser photocoagulation, and vitreoretinal surgery, but disappointing visual function, high anesthesia risk, eye infection, atrophia bulbi, limb necrosis and other complications are still troublesome[2]–[4], so energetically exploring better treatment and prevention measures is still necessary. The estrogen level is about 10 ng/ml in fetal blood, but it decreases rapidly after birth[5], so it is reasonable to hypothesize the cessation of exposure to estrogen of premature infants at birth may be related to some disorders that premature infants are prone. Beta estrogen possess a potent anti-oxidative function[6],[7], and oxidative stress plays an important role in the prognosis of retinopathy of prematurity[8]–[10], and beta estrogen receptor and the synthesis of 17 beta-estradiol (E2) have been found in retina[11],[12]. These results mean that beta estrogen may have an impact on ROP through an anti-oxidative pathway. So we design this experiment to verify this hypothesis.

## MATERIALS AND METHODS

### Animals

All experimental mice were neonatal C57BL/6J mice, purchased from Animal Experimental Center of Xian Jiaotong University. All animals were cared for in accordance with the Xian Jiaotong University Institute for Laboratory Animal Research (Guide for the Care and Use of Laboratory Animals) and the ARVO Statement for the Use of Animals in Ophthalmic and Vision Research.

### Reagents

E2, tamoxifen and fluorescein labeled high-molecular-weight dextran (2,000,000) were obtained from Sigma Corp., USA. The Mini malondialdehyde (MDA) assay kit was purchased from Biological and Engineering Institute of Nanjing Jiancheng, China.

### Model of oxygen induced retinopathy (OIR)[13]

Neonatal C57BL/6J mice was divided into six groups according to the treatment: room air with subcutaneous vehicle injection (control 1), hyperoxia with vehicle injection (control 2), hyperoxia with 0.1 µg E2 injection group, hyperoxia with 1.0 µg E2 injection group, hyperoxia with 10.0 µg E2 injection group, and hyperoxia with 10.0 µg E2 and 100.0 µg tamoxifen injection group. The pups received daily subcutaneous injections of either E2 in vehicle (dissolved in ethanol and diluted in 0.05 ml PBS) or vehicle alone from post-natal (p)7 to p17.The pups were exposed to 75% O_2_ from p7 to p12, along with nursing mothers. At p12, they were returned to room air and kept on feeding. The pups were killed on p17.

### Fluorescein-dextran perfusion of the retinal blood vessels and quantification of the avascular area

To study the retinal vascular pattern, retinal flat mounts were obtained by using perfusion of a high-molecular-weight dextran (2,000,000) conjugated with fluorescein (Sigma), as described previously[13],[14]. Briefly, the mice were anesthetized with intraperitoneal ketamine (60 mg/kg) and xylazine (18 mg/kg), a median sternotomy was subsequently performed. The left ventricle was identified, and 1 ml of a 50-mg/mL solution of high-molecular-weight fluorescein-conjugated dextran was injected with a 1-ml tuberculin syringe. The eyes were enucleated and placed in 4% paraformaldehyde in PBS. One hour later, the anterior segments were removed, the retinas with ora serratas intact were carefully dissected and placed into PBS. Four incisions were made in each retina, 90° apart, beginning at the ora serrate and extending centrally from the equator, stopping short of the optic nerve opening. The retinas were then placed onto a microscope slide and flattened a by coverslip and sealed with nail polish. Images of the superficial blood vessel layers were captured with fluorescence microscope (OLYMPUS BX-41, USA) in masked fashion and digitally stored for analysis.

### Analysis of peripapillary avascular areas

Digitized images of the total retinal area and the central capillary-free area was measured using Optimas software (Version 5.2).The central capillary-free area was expressed as a percentage of total retinal area.

### Pathological changes during revascularisation

During the phase of revascularisation, pathological vessel, neovascular tufts and clusters in retina appeared as described previously[15]–[17]. In flat mounts of different group of OIR mice at P17, the topographic distribution of neovascular tufts and clusters may not be uniform. However, as described by Lange *et al*[20], the whole retina can be subdivided by concentric rings into four zones, the distribution of tufts and clusters can be counted, and their percentage in different zones can be employed as indicators in describing the severity of OIR.

### Measurement of retinal lipid peroxidation

The lipid peroxidation concentration was determined by a method that measures the amount of thioibarbituric acid reactivity, which is expressed as the amount of MDA formed during acid hydrolysis of the lipid peroxide compound. Retinal tissue homogenates were prepared and reacted with sodium dodecyl sulfate, acetic acid, and thiobarbituric acid as described previously[18],[19]. Tetraethoxyopropane was used to establish a standard curve. The Bradford assay was performed to determine the protein concentration of retinal tissue lysate. Lipid peroxide level was expressed in terms of nmol MDA per mg protein.

### Statistical analysis

All data were expressed as mean ±SD. Differences among groups were analyzed by one-way analysis of variance (ANOVA), and the Student-Newman- Keuls (SNK) method was used for multiple comparison. The P-value reported was two-sided and value of *P* < 0.05 was considered statistically significant. All analyses were performed using the SPSS software (Version 11.0, SPSS Inc., USA)

## RESULTS

### E2 reduce retinal capillary-free area in OIR

**Fig. 1:**
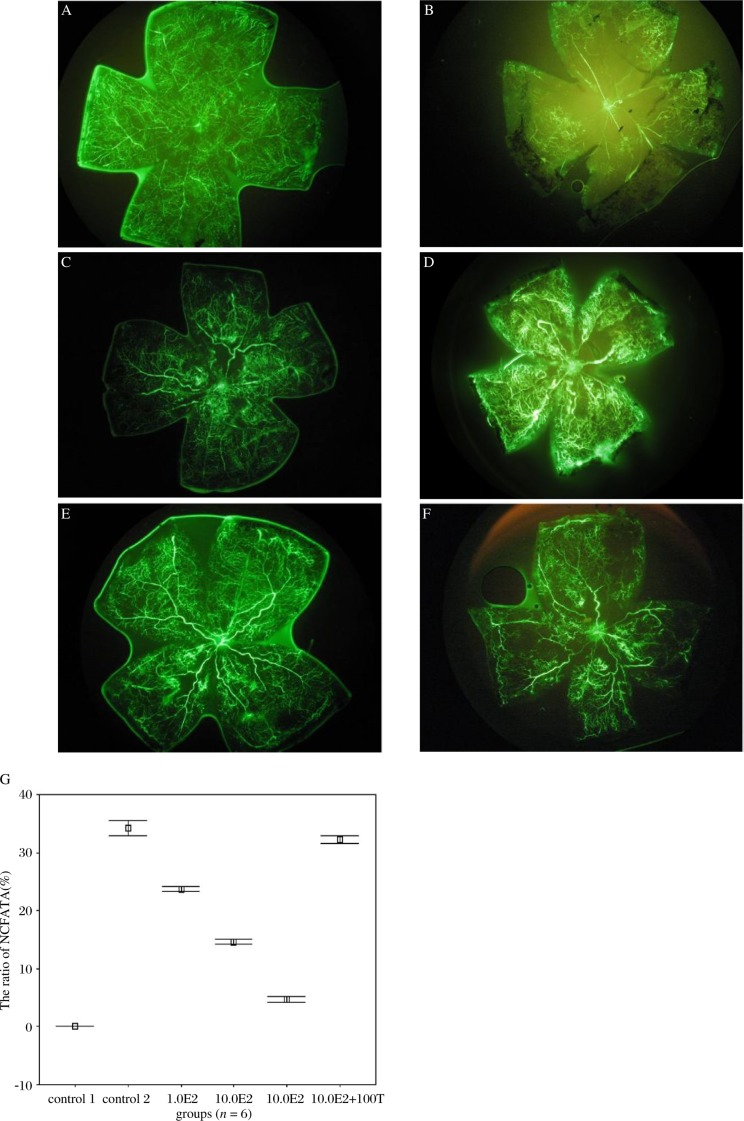
The effect of E2 against retina injury induced by hyperoxia exposed treatment A: There is no vascular free area in retina of control 1 group (×4). B: the presence of non-perfusion area in retina from mice of control 2 group (×4).C: 0.1 µg E2 reduced the non-perfusion area in retina induced by hyperoxia-exposed treatment (×4). D: 1.0 µg E2 further reduced the non-perfusion area in retina induced by hyperoxia-exposed treatment (×4). E: 10.0 µg E2 produced the maximal effect against the retinal injury induced by hyperoxia-exposed treatment (there is no significant difference between the effects of E2 at the concentration 100.0 µg with 10.0 µg, data are not shown) (×4). F: 100.0 µg tamoxifen blocked the protective effects of 10.0 µg E2 against retinal injury induced by hyperoxia- exposed treatment (×4). G: Mean ratio of NCFA/TA in groups with different E2 dose(presented as mean±SD). With the increase of E2 dose, NCFA/TA was decreased, and this function was reversed by tamoxifen (T).

In normal mice, capillary-free area was not found in retina (***Fig.1A***), while in mice exposed to hyperoxia (control 2), the ratio of Capillary-Free Area to Total Area (NCFA/TA) in retina is increased to 34.197±1.301% (***Fig.1B***). Treatment with E2 at the concentration of 0.1, 1.0, and 10.0 µg, decreased the ratio of NCFA/TA to 23.685±0.407 (*p* < 0.01 *vs* control 2, *n* = 6), 14.648±0.355 (*p* < 0.01 *vs* control 2, *n* = 6) and 4.693±0.450% (*p* < 0.01 *vs* control 2, *n* = 6) respectively (***Fig.1C-1E***, *n* = 6). 100.0 µg tamoxifen, a selective antagonist of estrogen receptor, significantly decreased the effect of 10.0 µg E2 on the ratio of NCFA/TA (***Fig.1F***, *n* = 6). These results (***Fig.1G***) suggested that activation of estrogen receptor produce retinal protective effect against oxygen induced retinopathy.

### Pathological changes during revascularization[20]

**Fig. 2:**
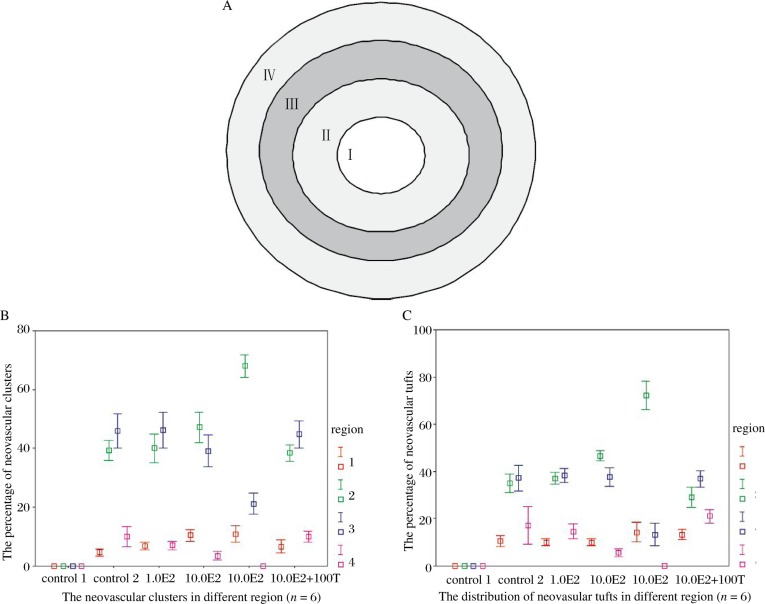
Pathological changes during revascularisation. A: the pattern of delimiting the whole retina into four zones (the distance among adjacent demarcation line is equal). B: the percentage of retinal neovascular clusters in different E2 groups at p17: in all groups. Most clusters were concentrated in region II and III, but not in region I and IV, and in 10.0 E2 group, more clusters were centralized in region II than other groups. C: the percentage of retinal neovascular tufts in different E2 groups at p17: in all groups. Most tufts were concentrated in region II and III, but not in region I and IV, and in 10.0 E2 group, more tufts were centralized in region II than other groups.

We also compared the changes of the neovascular tufts and clusters in the four zones of the retina (***Fig. 2A***). In contrast to normal mice, the number and percentage of neovascular tufts and clusters from zone I to IV of retina all significantly increased in hyperoxia-exposed mice. E2 caused a further dose-dependent increase in the percentages of neovascular tufts and clusters in region I and II (***Fig. 2B and 2C***). However, the percentage significantly decreased in region III and IV (*p* < 0.01 *vs* control 2, ***Fig. 2B and 2C***). In addition, the effect of 10.0 µg E2 was reversed by 100.0 µg tamoxifen. (clusters, *p* < 0.01 *vs* control 2; tufts: *p* < 0.01 *vs* control 2) (***Fig. 2B and 2C***).

### E2 significantly reduced the content of MDA in retina of hyperoxia-exposed mice.

**Fig. 3:**
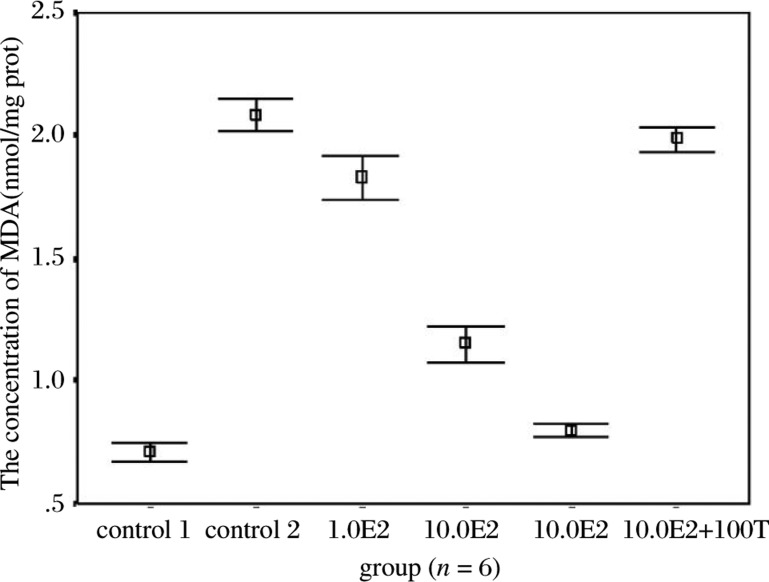
Mean retinal MDA concentration in groups with different E2 dose (presented as mean±SD). With the increase of E2 dose, MDA was decreased, and this function was reversed by tamoxifen (T).

Compared with control 1 group (0.711±0.037), the concentration of MDA in retina was significantly increased in hyperoxia-exposed mice in control 2 (2.084±0.066). Treatment with E2, at the concen- tration of 0.1, 1.0, and 10.0 µg, decreased the concentration of MDA to 1.829±0.091 (*p* < 0.01 *vs* control 2, *n* = 6), 1.152±0.067 (*p* < 0.01 *vs* control 2, *n* = 6) and 0.796±0.027 (*p* < 0.01 *vs* control 2, *n* = 6) respectively. 100 µg tamoxifen, a selective antagonist of estrogen receptor, significantly reversed the effect of 10.0 µg E2 on the concentration of MDA 1.988±0.049 (***Fig. 3***). Furthermore, there was a strong correlation between the changes of MDA and the protective effect of E2 (*F* = 628.103, *P* < 0.01). These results suggested that reduced free radicals formation may be involved in the protective effect of activation of estrogen receptor against hyperoxia-induced retina injury.

### Close relation between the percentage of retinal capillary- free area/total area and MDA

**Fig. 4:**
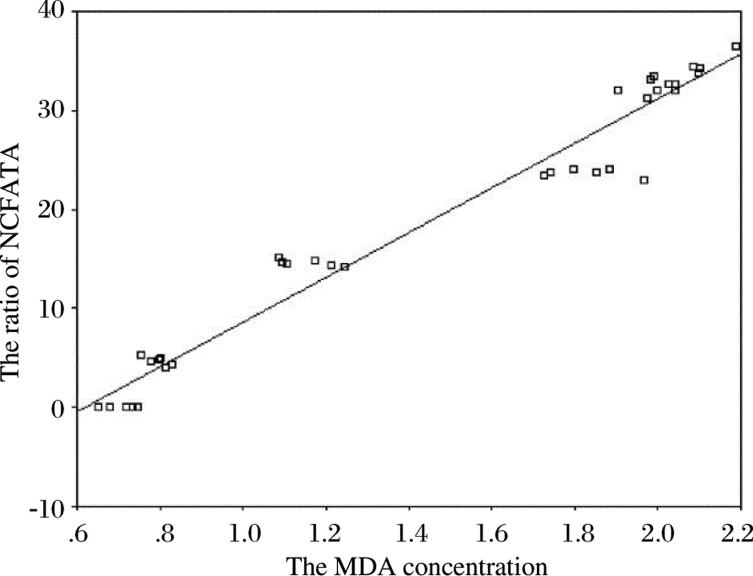
The relation between the ratio of Capillary free area /total area (NCFA/TA) and MDA. With the increase of MDA, NCFA/TA increased also, and they are highly correlated (ŷ = -13.954+22.57x, *r* = 0.981,*P* < 0.01).

In order to more easily understand the relation among retinal capillary- free area as a percentage of total area and MDA, the results were plotted and the trends between them was clearly manifested (***Fig.4***): The retinal avascular area as a percentage of total area was changed in the MDA concentration. When MDA concentration increased, the percentage of avascular area increased, and vice versa. These results mean that avascular area was highly correlated with MDA concentration (*r* = 0.981, *P* < 0.01).

## DISSCUSION

By determining the differences in the percentage of avascular area/ total retinal area in different groups, we can understand the effect of E2 on retinal angiogenesis of OIR mice. The retina, extremely rich in membranes with polyunsaturated lipids[21], possessing a high metabolism rate, high oxygen consumption and an imperfect scavenger system for the products of oxidative stress in premature infants, is susceptible to oxidative damage[25]–[28]. In this study, a small amount of MDA was found in normoxia retina. After the stimulus of hyperoxia, the quantity of MDA increased significantly (*t* = 49.059, *P* < 0.01). With administration of E2, the increase of MDA was reduced in a dose-dependent manner, and an estrogen receptor antagonist reversed this action. Thus our data suggest that a certain amount of oxidative stress reaction may contribute to retinal angiogenesis, while excessive oxidative stress has the opposite effect. Through its receptor pathway, E2 can alleviate the excessive oxidative stress response, and finally ameliorate retinal angiogenesis. In hyperoxia, the percentage of retinal avascular area was not further improved with an increase in the E2 dose above 10.0 µg (results not shown here), indicating that the OIR can not be completely eliminated by E2. Therefore other elements must be involved in this pathological process, which need to be further explored.

Another study demonstrated that retinal angiogenesis could be modulated by E2 through HIF-1 alpha and VEGF pathway, and thus improve OIR[29].In the present study, increasing the E2 dosage, decreased the MDA concentration. This means that E2 can exert its protective function not only through HIF-1 alpha and VEGF pathway, but also by altering the oxidative stress response[10],[25], but the intrinsic relations between oxidative stress and VEGF may not be negligible factor[30],[31].

Altogether, oxidative stress response may play a pivotal role in OIR through receptor pathways, E2 can mitigate the injury of excessive oxidative stress response in oxygen exposed retina, and thus ameliorate OIR. Further more, the results of this study suggest that fully studying the change of endocrine hormone in pregnancy and fully understanding their function in fetal tissue development, may provide better prophylaxis for ROP. Recently, it has been reported that the fragment of prolactin (17-PRL) exerts more powerful anti-angiogenesis[32],[33], and it may be employed as another important hormone in the prophylaxis of ROP.

The mechanism of antioxidative stress is complicated *in vivo*. The pathways related to estrogen include P13K/Akt-Nrf2 signaling pathway[34],[35],MAPK(MEK/ERK1/2) pathway[36],[37], and the mechanism of estrogen mediated protection in this pathological process need to be further studied.
